# Optimizing Endoscopic Respiratory Diagnostics with Cytology: An Update on Touch Imprints with a Comparative Literature Review

**DOI:** 10.3390/diagnostics14232750

**Published:** 2024-12-06

**Authors:** Hatice Elmas, Binnur Önal, Selda Yilmaz, Stefan Steurer, Lutz Welker

**Affiliations:** 1 Section Cytopathology, Institute of Pathology, University Medical Center Hamburg-Eppendorf UKE, D-20246 Hamburg, Germany; s.steurer@uke.de (S.S.); l.welker@gmx.net (L.W.); 2 Central Pathology Laboratory, Acıbadem Healthcare Group, Acıbadem University, Atasehir, 34752 Istanbul, Türkiye; binnurtr@gmail.com; 3 LungenClinic Grosshansdorf, D-22927 Grosshansdorf, Germany; yilmaz.selda@yahoo.de; 4 Airway Research Center North (ARCN), German Center for Lung Research (DZL), D-35037 Marburg, Germany

**Keywords:** lung cytology, touch imprint, scrape, endoscopic biopsy, cytopathology, diagnostics, optimization

## Abstract

**Background:** Major diagnostic and therapeutic changes led to the implementation of the ‘lung cancer diagnosis in small biopsies and cytology specimens’ concept in the WHO Classification of Tumours of the Lung, Pleura, Thymus and Heart in 2015. Touch imprints are an established technique in cytology that provides a rapid and minimally invasive method for morphological diagnoses of clinical specimens, accurate subtyping, and molecular characterizations of malignancies. The extended diagnostic requirements from the increasingly limited material provided by minimally invasive biopsy techniques pose major challenges for pathology. Recognizing the relevant features and potential pitfalls is essential for cytologists to avoid misinterpretation. **Materials and Methods:** A retrospective analysis of endoscopic and surgical biopsy diagnostics was performed on 717 patients (303 women and 414 men; average age of 66.9 years) with clinically suspicious tumor findings at the LungenClinic Grosshansdorf in 2020. A total of 1363 cytological samples were obtained pre-therapeutically from 986 distinct biopsies covering 330 surgically and 656 endoscopically examined pulmonary, mediastinal, and bronchial regions. These samples were then compared with the histological diagnoses that were critical for determining the final therapy. **Results**: Out of a total of 656 endoscopically examined cases, 322 were classified as malignant, 308 as benign, and 26 as undetermined. While touch imprints and histological analysis separately achieved specificity values of 95.4% and 98.8%, both methods showed sensitivity values of 82.1% and 86.5%, respectively. In contrast, combining the two methods improved the sensitivity by 8 percentage points to 94.6%. Out of 330 cases of surgically examined samples, 137 were malignant, 190 were benign, and 3 were undetermined. The specificity of the morphological examinations for these samples was comparably high at 97.9% and 100%, respectively. In this surgical setting, touch imprints alone achieved a sensitivity of 75.9% (n = 104/137 cases), with a specificity of 97.9% (n = 186/190 cases). The outcome of the histological approach alone and in combination with touch imprints reached a sensitivity of 96.4% (n = 132/137 cases). **Conclusions:** Cytology and histology achieved comparably high sensitivity and specificity values on small biopsies. Under optimal conditions for morphological analysis in a surgical setting, the sensitivity of cytology for detecting malignant tumors was only 6 percentage points lower compared with the clinical endoscopic setting. A combined approach of cytologic–histologic evaluation for endoscopically examined specimens significantly increased the sensitivity by approximately 8% compared with the surgical setting (*p* < 0.003).

## 1. Introduction

No art or science is eternally valid. If essential conditions change, even seemingly or actually, golden rules must be reviewed. This applies to cytology and histology. The development of endosonography and positron emission tomography, flexible bronchoscopes and biopsy forceps with decreasing diameters, molecular pathology, and the introduction of targeted therapy have permanently changed both the biopsy material and the spectrum of patients. These fundamental changes have led to the application of the concept of ‘lung cancer diagnosis in small biopsies and cytology specimens’ in the WHO 2015 Classification of Tumours of the Lung, Pleura, Thymus and Heart [1]. 

Touch imprints provide an accurate, rapid, and minimally invasive method for assessing the cytopathological diagnosis of clinical specimens, subtyping, and molecular characterization of malignancies [2,3,4,5]. The method’s simplicity, combined with its ability to reveal characteristic cytomorphological features, makes it an indispensable tool, even in modern diagnostic cytology [6,7].

Touch imprint cytology is emphasized in specific clinical contexts, showcasing its utility in various diagnostic and intraoperative settings. For instance, in the diagnosis of endoscopically nonvisible lung malignancies, the integration of touch imprint cytology with rapid on-site evaluation (ROSE) significantly improves diagnostic accuracy and efficiency [8,9,10].

Bronchoscopic biopsy diagnostics aim to identify bronchopulmonary lesions. Since the 1990s, all types of conventional cytology techniques have been reported to enhance the diagnostic value of cytopathology in lung cancer efficiently using a combined approach [11,12]. 

Reviewing the importance of classical morphology in the future of cytopathology, its scope is expanding. Classical cytomorphology has essential importance for diagnosis and improving our understanding of cancer biology. New endoscopy and imaging techniques are replacing surgical biopsies with cytology samples. New molecularly targeted therapies allow cytology to play a major role, having the ability to achieve cost-effective healthcare outcomes; however, they pose new challenges [8].

In the case of malignant tumors, this diagnosis aims to obtain prognostically and therapeutically relevant information on tumor size and expansion as well as lymph node status [12,13]. The management process of patients with bronchopulmonary lesions alongside lymph node enlargement also covers the differential diagnosis of tumors from upper respiratory tract and lung infections causing necrotizing granulomatous lymphadenitis, including pulmonary tularemia [14].

Recent technical developments and findings have changed modern bronchoscopic diagnostics [15]. Differentiated biopsy techniques and ancillary procedures have considerably expanded the diagnostic spectrum [3]. However, changes in the quality and quantity of biopsies and the questionable differential therapeutic relevance of subtle histological type and subtype diagnoses have led, in practice, to a higher percentage of bronchial carcinomas being diagnosed cytologically [16,17]. 

Moreover, this technique holds significant academic and clinical merit due to its immediate diagnostic capabilities, which are pivotal for intraoperative consultations, rapid remote online evaluations, and prompt therapeutic interventions [4,17,18,19,20,21,22]. 

The quality of these imprints is often comparable to more complex sampling methods, making touch imprints a favored choice for pathologists in various scenarios [17,18,21]. 

However, despite their advantages, touch imprints come with their own set of limitations and potential pitfalls that must be navigated carefully to ensure accurate interpretations [10,17]. Concerning the implementation of lung cancer diagnosis in small biopsies and cytological samples, this study aimed to answer the following questions:

1. What is the diagnostic performance of each separately and a combined approach of imprint/scrape cytology and histopathological examination for endoscopic and surgical biopsies compared with the final histological diagnosis?
2. Are there any added values of the combined touch imprint cytology–histology approach for endoscopic and surgical biopsies in comparison?

## 2. Materials and Methods

**Figure 1:**
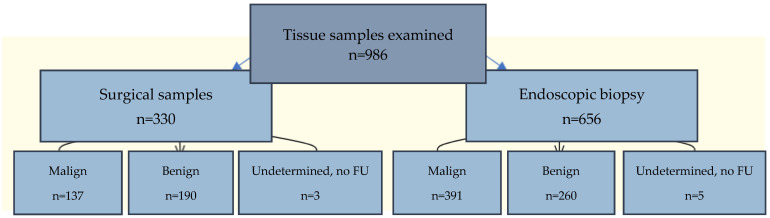
This flowchart represents the distribution of tissue samples (n = 986 biopsy tissues/gender–age distribution: F = 303, average age = 66.1; M = 414, average age = 67.5) into two categories: surgical (n = 330) and endoscopic (n = 656) tissues. These categories are further subdivided into malignant, benign, and undetermined cases. FU: follow-up.

A retrospective analysis of endoscopic and surgical biopsy diagnostics was performed on 717 patients (303 women and 414 men; average age of 66.9 years) with clinically suspicious tumor findings at the LungenClinic Grosshansdorf in 2020. A total of 1363 cytological specimens were collected from 986 biopsy tissues, which included both surgically (330) and endoscopically (656) examined pulmonary, mediastinal, and bronchial regions. Duplicates were identified and excluded. Some repeat samplings were conducted to confirm diagnoses that were not included in the diagnostic performance calculations. The cytological findings were then compared with the corresponding histological diagnoses, which were integral to determining the final therapeutic approach (Figure 1).

In this context, the lack of evidence of malignant changes in lymph nodes, the bronchial wall, or different lung areas is particularly important for the pre-therapeutic detection of tumor spread and the classification of the tumor stage. 

The collection process was performed following ethical guidelines and with each patient’s informed consent. All patient data were anonymized to ensure confidentiality. The data were then coded and entered into a secure database for subsequent analysis. 

Samples were collected from patients diagnosed with bronchopulmonary lesions during routine clinical assessments at LungenClinic Grosshansdorf.

In detail, one to two impression preparations of bronchoscopically endoscopic biopsies were prepared by assistant personnel, and one to four scrapings of intraoperative surgical biopsies (wedge biopsy and lymph nodes) were prepared and stained with standard MGG (Giemsa) stain. 

For endoscopic samples, the assistant staff or clinicians routinely sent one and occasionally two samples. However, in the case of wedge biopsies, the cytologist usually prepared four scrapings. The routine staining, the final cytological diagnosis, and the comparison with other results of all further cytological/histological analyses were carried out at the Institute of Pathology of the University Medical Center Hamburg Eppendorf (UKE), Hamburg.

The data were then coded and entered into a secure database for subsequent analysis. The cytological tumor typing was based on the recent WHO classification for thoracic tumors [23] and the WHO 2022 Reporting System for Lung Cytopathology [24].

**Statistics:** This study conducted a retrospective analysis of UKE’s cytological data and electronic records, assessing touch imprints of both endoscopically and surgically acquired specimens in addition to clinical endoscopic findings and diagnoses, as well as patient follow-up data. Additionally, the analysis aimed to determine the sensitivity, specificity, and positive and negative predictive values. Fisher’s exact test was utilized to analyze the data, and all *p*-values of less than 0.05 were considered significant.

## 3. Results

This study conducted an analysis using both surgically and endoscopically acquired touch imprints to evaluate their characteristics. A total of 1363 cytological examinations of 986 different biopsy tissues were performed, with 330 being intraoperative and 656 endoscopic (Figure 1). The patient demographics consisted of 717 individuals, with 303 females and 414 males, with average ages of 66.1 and 67.5 years, respectively. The overall average age of the patients was 66.9 years. These data reflect the distribution and utilization of touch imprints in this study, highlighting the method’s application across different patient demographics.

The 986 biopsied tissue sections comprised 528 malignant and 450 benign lesions. In eight cases, it was not possible to determine the diagnosis with certainty. Three of these were surgical specimens (a cilium-bearing muconodular papillary tumor of the lung of unclear diagnosis, atypical adenomatous hyperplasia with a transition to adenocarcinoma in situ, and a lymph node fragment, in which evidence of a marginal-zone lymphoma was detected after 2021), alongside five endoscopic biopsies with suspected histological tumors without any follow-up. 

**Figure 2:**
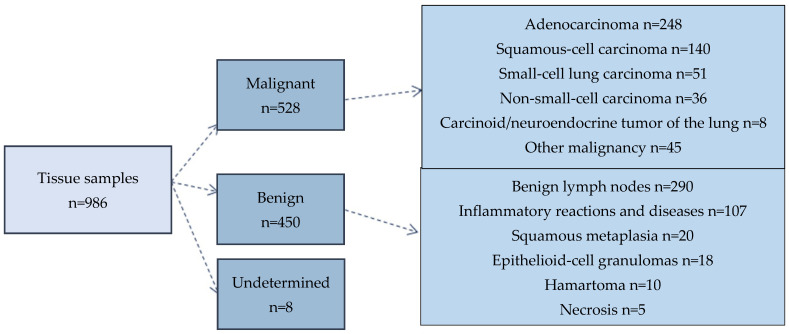
The flow chart categorizing the data from surgical and endoscopic acquisition and final histological types.

Of the 528 malignant tumors, 483 were lung carcinomas, and 45 were other malignant tumors (thymomas, melanomas, and other sarcomas, as well as malignant lymphomas). Benign lesions were present in 450 cases (such as normal, reactive, and inflammatory lymph node lesions; inflammatory, granulomatous, and metaplastic reactions; and benign tumors, such as hamartomas) (Figure 2).

First, the effectiveness of touch imprints alone on endoscopically achieved biopsy material was analyzed (Table 1). Out of 656 cases, 322 were classified as malignant, 308 as benign, and 26 as indeterminable (due to technical and qualitative reasons). Malignant cases predominated (n = 391). Benign cases detected as part of the spread diagnosis were also significant (n = 260). The number of undetermined cases was comparatively low (n = 5; sensitivity 84.9%; specificity 100.0%). 

Second, the outcome of histological diagnosis alone on endoscopic biopsy material was analyzed. Among the 656 cases examined, 338 were identified as malignant, 297 as benign, and 21 as undetermined (sensitivity 89.4%; specificity 100.0%) (Table 1). There were no significant differences between touch imprint and histological diagnosis alone (*p* > 0.072).

Furthermore, the diagnostic outcome of the combined approach of touch imprint and histological examination for endoscopic biopsy material was analyzed (Table 1). Out of the 656 cases examined, 391 were identified as malignant, 260 as benign, and 5 as undetermined. Malignant cases comprised the majority (n = 371), with a higher number detected using combined touch imprint and histology compared with either method individually. Benign cases were also notable (n = 260), suggesting the efficacy of the combined approach in accurately diagnosing non-malignant lesions (sensitivity 95.6%; specificity 100.0% (Table 1)). There were highly significant differences between touch imprint and histological examination alone and the combined analysis (*p* < 0.003 and *p*< 0.0001, respectively). 

In addition, the results of the morphological analysis of surgically acquired material were investigated. Out of the 330 cases evaluated, 104 were malignant, 213 were benign, and 13 were undetermined (Table 2). In this surgical setting, touch imprints alone achieved a sensitivity of 80% (104 of 137 cases), with a specificity of 100% (104 of 104 cases (Table 2)). The outcome of the histological approach alone and combined with touch imprints reached a sensitivity of 97.1% (132 of 137 cases) and a specificity of 100% (132 of 132 cases (Table 2).

## 4. Discussion

In this study, the results of the cytological–histological examinations of the endoscopic biopsies were very credible, with specificity values of 95.4%, 98.8%, and 94.2%, respectively. Regardless of this, malignant tumors were detected with sensitivities of 82.1% and 86.5% with cytological and histological examinations, respectively. Combining the methods increased the sensitivity to 94.6%. 

Endoscopic techniques can become more widely used for tumor evaluation, monitoring, and assessing treatment responses. Tissue sampling should be maximized whenever feasible and deemed clinically safe to allow for extended diagnostic and predictive examinations, reducing the need for re-biopsy for additional studies, and tissue handling, processing, and sectioning should be optimized [25].

The WHO classification does not apply in full to small tissue biopsy or cytology samples; nonetheless, virtually all patients with a tissue diagnosis of lung cancer are initially diagnosed based on such small specimens. In most of these cases, there is no subsequent surgical resection to allow full confirmatory diagnosis. The 80% of NSCLC patients with advanced disease receiving chemotherapy have only small biopsy and/or cytology samples available for diagnosis [23,26]. Pathologists accept that a specific diagnosis cannot be made in some cases because objective features are not present in the cytological and/or histological specimens. From this point of view, the relevant differences between touch imprints intraoperatively become understandable to pathologists (Table 3).

Simultaneously, introducing targeted therapies for advanced lung cancer has significantly increased the number of these patients and broadened the spectrum of biopsy to include other metastatic tumors. In such cases, this extended diagnostic requirement from increasingly limited material provided by minimally invasive biopsy techniques poses major challenges for pathology [26,27].

In contrast with surgical biopsy, combined cytological–histological evaluations of endoscopic samples lead to a significant increase in sensitivity of approximately 8%.

Kops et al. reported a sensitivity of 84.4% and a specificity of 99% for touch imprints in the evaluation of small lung nodules during navigational bronchoscopy [28]. In our study, the sensitivity for endoscopic biopsies was 82.1%, which is consistent with Kops’ findings, demonstrating the reliability of touch imprints as a minimally invasive diagnostic method. Both studies emphasize the utility of touch imprints for rapid and accurate diagnosis, particularly in cases where small biopsy samples are used. 

Similarly, Muto et al. assessed touch imprints combined with rapid on-site evaluation (ROSE) for peripheral pulmonary lesions and reported a specificity of 90% and a sensitivity of 69% [9]. While the specificity is comparable to our results, our study found a higher sensitivity, possibly reflecting differences in patient selection or sample preparation techniques. 

Padmanabhan et al.’s survey on touch imprint cytology during needle core biopsies reported similar specificity (100%) but slightly higher sensitivity (89%) [10]. These results, supporting our study, highlight the reliability of touch imprint-based methods, particularly when combined with histology and addressing the advantages of rapid intraoperative techniques, like ROSE, while benefiting from the precision of controlled laboratory conditions.

Hantera et al. reported a sensitivity of 94% and specificity of 100% for touch imprints in lung and pleural lesions [29]. This sensitivity is significantly higher than the rate of 82.1% sensitivity observed in our study for endoscopic biopsies. This discrepancy may be attributable to differences in biopsy techniques or the quality of cytological samples. Hantera’s study involved multiple biopsy samplings, which might inherently yield higher sensitivity compared to endoscopic biopsies. 

Botticella et al. reported a sensitivity of 77% for touch imprints during bronchoscopy, which is slightly lower than our findings [16]. This difference may be due to variations in procedural expertise or differences in patient cohorts. In our study, the use of combined cytological–histological evaluation significantly improved sensitivity (94.6% for endoscopic biopsies) [16]. This improvement emphasizes the complementary nature of these methods, particularly when the sample size or quality is limited. The variability in sensitivity across studies suggests that touch imprint outcomes can be influenced by factors such as operator experience, preparation techniques, and sample quality. 

**Figure 3:**
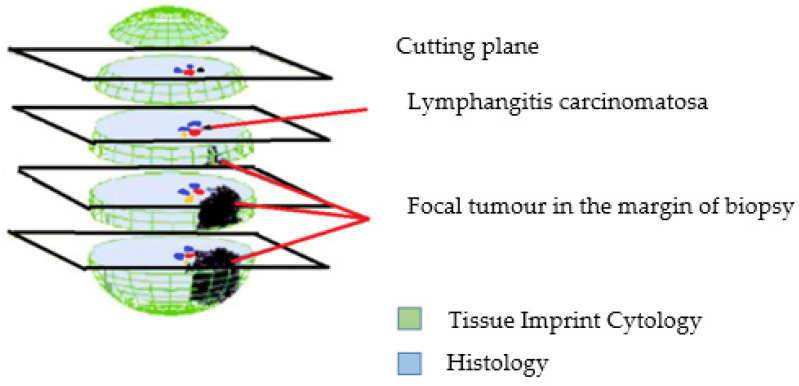
(Modified from Ref. [21]): A visual representation depicting the factors contributing to undetermined findings between touch imprints and histology in paraffin sections. Despite conducting serial sections, localized tumor areas previously detected via touch imprints may go unnoticed during histological examinations. In such scenarios, if touch imprints yield positive results, the comprehensive processing of the biopsy with numerous serial sections becomes imperative.

A tissue sample is typically embedded in paraffin for histological analysis. During this process, the tumor tissue can be located in the deeper layers of the sample, which may not be immediately visible when examined macroscopically. Therefore, the tumor may not be detected in the initial sections taken from the sample because it is situated deeper within the paraffin block. However, it might be possible to observe the vessels under the microscope as the sectioning process continues, even indicating the presence of tumor tissue pointing to lymphangitis carcinomatosa. If these vessels contain tumor cells, invasive growth is indicated, although this may not be detectable in each section (Figure 3).

The tumor may eventually be revealed as deeper sections are examined. The diagnostic yield of histology depends on the planes in which these sections are taken. If the tissue sample is rolled or moved onto the slide before being embedded in paraffin, different portions of the tissue’s surface will be transferred onto the slide. This means that the surface, which might be tumor-free, alongside deeper tissue layers, can be examined cytologically (Figure 3).

However, the disadvantage of cytology is that it cannot detect invasive growth in blood vessels. The larger the tissue, the greater the risk of missing the tumor in different section levels. This implies that the larger the tissue sample, the relatively fewer sections are made, increasing the risk of a missed histological diagnosis. As the biopsy size decreases, the discrepancies between cytology and histology diminish. In endoscopic biopsies, it is more likely that histological sections will capture the tumor tissue and that cytology and histology will be consistent with each other and enhance the diagnostic capacity (Figure 3).

In addition, the sensitivity of histological examinations decreases in any situation where there is a quantitative disproportion between the incised tissue and the tissue embedded in the paraffin block. This is particularly relevant in large endoscopic biopsies, where a significant portion of the tissue might not be adequately represented in the sections. In such cases, combining cytological and histological examinations will likely result in a greater increase in diagnostic sensitivity compared with small samples that are completely processed. This combined approach helps to ensure that one method may still detect the tumor even if the other misses it, thereby improving the overall diagnostic accuracy (Figure 3) [21].

Overall, while touch imprints offer several advantages in terms of rapid diagnosis and minimal sample processing, they also have limitations that must be considered when interpreting cytological findings and making clinical decisions. Table 3 provides a more comprehensive overview of the advantages and disadvantages of touch imprints.

**Table 3:** The pros and cons of touch imprints in the literature.

Advantages	Detailed Explanation		Ref.
Provide rapid diagnosis	TPs offer quick insights into tissue samples and are particularly valuable in surgical settings or urgent diagnostic scenarios.		[9,17,28,30]
Require minimal processing	TPs involve minimal processing steps, reducing analysis time and minimizing processing artifacts.		[10,31]
Preserve cellular morphology	TPs retain cellular morphology well, allowing visualization of crucial cytological features.		[21,32,33]
Suitable for small samples	TPs are useful for small or fragmented tissue samples, enabling assessment without extensive handling.		[10,17,21]
Enable real-time evaluation	TPs allow real-time evaluation, providing immediate feedback to clinicians and influencing decision-making.		[2,34,35]
Minimally invasive, rapid diagnosis	TPs preserve cellular morphology for accurate and rapid diagnosis of small biopsies.		[17,36,37,38,39]
Well-suited samples at molecular level	TPs reduce amount of connective tissue and increase amount of fully intactnuclei in tumor cells.		[3,15,20]
**Disadvantages**	**Detailed explanation**	**Solutions**	
Sample only tissue surface	TPs collect cells only from the surface of the tissue, potentially missing deeper pathological features or structures crucial for accurate diagnosis.	***Combine with other techniques:*** Use TPs in conjunction with deeper tissue sampling techniques, such as biopsies, to ensure comprehensive analysis. Develop imaging technologies that can penetrate deeper tissue layers for more thorough examinations.	[17,21,39]
Risk of artifacts, such as crushing or contamination	Despite minimal processing, TPs are still susceptible to artifacts, such as cell crushing, distortion, or contamination from adjacent tissues, affecting diagnostic accuracy.	***Implement quality control measures:*** Establish rigorous quality control measures to detect and prevent artifacts. Utilize appropriate techniques for sample collection and processing to minimize the risk of contamination or distortion.	[10,18,35,40]
Interpretation can be subjective	Interpretation of TPs depends heavily on the experience and expertise of the cytotechnologist or pathologist, introducing subjectivity and potential variability in diagnosis.	***Utilize consensus review:*** Implement consensus review practices where multiple experts review challenging cases to minimize subjective interpretation and improve diagnostic accuracy.	[21,32,41]
Limited ability for additional testing	TPs may not always provide sufficient material for ancillary testing, such as immunohistochemistry or molecular analysis, which can be crucial for confirming diagnoses.	***Integrated approach:*** Combine TPs with other sampling methods or techniques to ensure an adequate supply of material for additional testing. Develop innovative technologies for on-site ancillary testing to supplement TPs and enhance diagnostic capabilities.	[17,42]
Possibility of false negatives due to limited sampling	Due to their limited sampling depth and potential for artifacts, touch imprints may yield false-negative results, particularly in cases where target cells are sparse or obscured.	***Exercise caution in interpretation:*** Exercise caution when interpreting TPs, especially in cases where results are inconclusive or contradictory. Consider complementary diagnostic approaches to confirm findings and minimize the risk of false negatives.	[17,43]

Abbreviations: Ref., reference; TP, touch imprint cytology preparations.

Minimal processing steps, short turnaround times, and the ability to analyze in real time are key advantages of touch imprints. Closely linked to this is the ability to compensate for relevant errors in sample collection. This is particularly advantageous for the rapid assessment of bone tumors, as no decalcification is required [44]. Recognized for its simplicity, touch imprinting entails cellular collection from tissue samples by pressing them onto a slide, typically from core biopsies or fresh surgical specimens. Subsequent staining of these slides reveals intricate cellular structures and arrangements, offering cytologists valuable insights. Often, the quality of these impressions is remarkably high, rendering touch imprints a preferred option for pathologists across diverse clinical contexts [36,40,42,44,45].

In addition, this technique is particularly valuable for its clinical utility in providing immediate diagnostic insights, which can be crucial for intraoperative consultations and swift therapeutic decisions [34,46]. 

Intraoperative consultation cytology also includes crushing (squash preparation) in addition to traditional impression cytology, such as imprinting, scraping, and spreading. It is often used for the evaluation of the surgical margins of bronchopulmonary mass lesions and lymph nodes for staging. Using scraping cytology to evaluate the surgical margins in a patient operated for bronchial carcinoma may often display the carcinoma cells and even the chondroid tissue, with the quality indicator reflecting the sampling accuracy [47].

In addition, touch imprints are well suited and promising for remote rapid on-site evaluations (ROSE) in modern applications [9,17,32,34]. This method allows for the immediate assessment of biopsy samples, enhancing the ability to identify malignancies that are not visible endoscopically. The findings indicate that the diagnostic yield of transbronchial biopsy for such malignancies is markedly improved when touch imprint cytology is employed alongside ROSE, demonstrating the method’s vital role in real-time clinical decision making [9,37,48].

Touch imprint cytology was also used in the Telecytology and Tele_Rose studies, providing the rapid evaluation of one glass slide by several users in a synchronized or asynchronized manner and without any loss of technical quality [4,42]. This is a major advantage of digital image transferring and storage.

The integration of touch imprint cytology with other diagnostic and surgical techniques enhances the precision and efficiency of cancer diagnosis and treatment. Its ability to provide rapid, on-site evaluations makes it an essential component of modern oncology practices for various organ systems, enabling clinicians to make informed decisions swiftly and improve patient outcomes [22,30,34,36,42,43,44,45,48]. 

As in clinical cytology, imprint and scrape cytology techniques are performed in the autopsy suite. Imprinting and scraping have been primarily used in conventional autopsies [49]; however, also in virtual autopsies, they are used to detect preliminary diagnoses in many parenchymatous organs, including lung and mediastinal lymph nodes, and for the training of residents [50]. 

In terms of quality assurance, some technical issues can affect sensitivity, including artifacts, such as cell crushing, distortion, or contamination from adjacent tissues, which can affect diagnostic accuracy. The method also has limitations in terms of the sampling depth, amount of material, and number of ancillary tests. Regarding cell morphology, the method allows for a well-preserved morphology and detailed recognition of cell types, architectures, and growth patterns as long as principles and specific guidance is performed for validating immunohistochemistry on cytology specimens [51].

**Figure 4:**
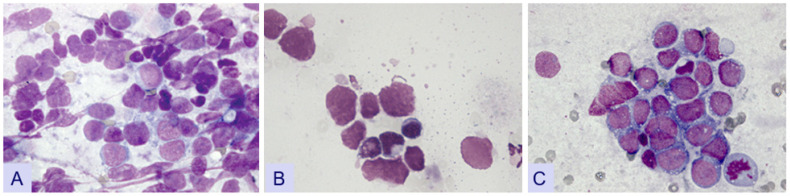
(**A**): Diagnostic difficulties caused by excessive pressure in touch imprint cytology (SCLC) (Giemsa 640×); (**B**): swelling artifacts (Giemsa 640×); (**C**): NHL (Giemsa 640×).

Various errors can occur during the preparation of cell smears, impacting sample quality (Figure 4). Several pre-analytical factors may influence the reliability of ancillary techniques. A common mistake is failing to spread the material on the labeled side of the slide, leading to material loss, particularly during wiping after staining. Another error is applying excessive pressure during spreading, resulting in crushing artifacts and affecting the cell morphology [17]. Furthermore, selecting too brief a drying time can cause material drift and compromise the smear quality. Recognizing these potential sources of error is crucial, prompting the implementation of appropriate measures to ensure the quality of cell smears.

Additionally, the aim is to offer comprehensive guidance for optimizing the diagnostic process by addressing the selection of ancillary tests most suitable for the predicted diagnosis [52]. However, the experience and expertise of the cytotechnologist and/or pathologist are critical. A lack of familiarity with the correct preparation and interpretation of touch imprint slides may be a disadvantage. 

Cytological results should be integrated with histological and ancillary, molecular test results for a comprehensive diagnosis. Overall, Table 3 emphasizes the importance of experience and expertise in the field of cytopathology for optimizing endoscopic respiratory diagnostics by Touch Imprint Cytology. 

Cytological samples represent a valid source of high-quality RNA and DNA for NGS analysis, especially for predicting patients’ response to targeted treatments and for refining the risk of malignancy in indeterminate cytological diagnoses. The methodologically uncut, fully intact tumor cell nuclei in low-stroma monolayer cytology preparations are ideal for complementary molecular pathological analyses (FISH and NGS), especially when the paraffin blocks contain only sparse tumor sections [15,53,54]. The diversity of cytopreparations and the resource-intensive clinical validation of NGS pose significant results to more consistent use of non-cell block cytology specimens, including imprint smears [55].

## 5. Conclusions

Minimally invasive cytological procedures, such as touch imprints, facilitate comprehensive tumor assessments and treatment response monitoring. These methodologies are instrumental in assessing the efficacy of targeted therapies by elucidating molecular tumor profiles and gauging treatment outcomes. Consequently, touch imprints could potentially mitigate the need for surgical interventions while fostering the prominence of endoscopic procedures in targeted cancer treatment [17,56].

Cytology and histology achieve comparably high sensitivity and specificity values in small biopsies. Under optimal conditions for morphological analysis in surgical samples, the sensitivity of cytology for detecting malignant tumors is only slightly lower than that of endoscopic biopsy by approximately 6 percentage points. On the other hand, a combined approach of cytologic–histologic evaluation for endoscopic specimens leads to a significant increase in sensitivity of approximately 8% (*p* < 0.003). 

## Figures and Tables

**Table 1:** Comparison of touch imprint cytology and endoscopically acquired specimens with the final histological diagnosis (n = 656).

Diagnostic Technique		Final Histological Diagnosis				Sensitivity, Specificity, PPV, NPV, Diagnostic Accuracy
Cytological diagnosis (touch imprints)		Malign	Benign	Undetermined	Total	82.1%, 95.4%, 96.4%, 78.0%, 86.7%
	Malign	321	0	1	322	
	Benign	57	248	3	308	
	Undetermined	13	12	1	26	
	Total *	391	260	5	656	
Histological diagnosis		Malign	Benign	Undetermined	Total	86.5%, 98.8%, 99.1%, 82.9%, 90.7%
	Malign	338	0	0	338	
	Benign	40	257	0	297	
	Undetermined	13	3	5	21	
	Total *	391	260	5	656	
Combined cytological–histological diagnosis		Malign	Benign	Undetermined	Total	94.6%, 94.2%, 96.1%, 92.1%, 93.7%
	Malign	370	0	1	371	
	Benign	17	245	0	262	
	Undetermined	4	15	4	23	
	Total *	391	260	5	656	

Abbreviations: PPV, positive predictive value; NPV, negative predictive value. * Number of specimens.

**Table 2:** Comparison of touch imprint cytology and surgically acquired specimens with the final histological diagnosis (n = 330).

Diagnostic Technique		Final Histological Diagnosis				Sensitivity, Specificity, PPV, NPV, Diagnostic Accuracy
Cytological diagnosis (scrape and/or touch imprints)		Malign	Benign	Undetermined	Total	75.9%, 97.9%, 96.3%, 84.9%, 87.9%
	Malign	104	0	0	104	
	Benign	26	186	1	213	
	Undetermined	7	4	2	13	
	Total *	137	190	3	330	
Histological diagnosis		Malign	Benign	Undetermined	Total	97.1%, 100.0%, 100.0%, 97.9%, 97.9%
	Malign	133	0	0	133	
	Benign	4	190	0	194	
	Undetermined	0	0	3	3	
	Total *	137	190	3	330	
Combined cytological–histological diagnosis		Malign	Benign	Undetermined	Total	96.4%, 97.9%, 100.0%, 97.4%, 96.4%
	Malign	132	0	0	132	
	Benign	5	186	0	191	
	Undetermined	0	4	3	7	
	Total *	137	190	3	330	

Abbreviations: PPV, positive predictive value; NPV, negative predictive value. * Number of specimens.

## Data Availability

The data that support the findings of this study are available from the corresponding authors upon reasonable request.
